# A radiation-free novel approach for intestinal stent placement: the “scope-in-scope” technique

**DOI:** 10.1055/a-2324-2108

**Published:** 2024-07-30

**Authors:** Jiaqing Hu, Jinhui Zheng, Changshun Yang, Xiang Gao, Xianbin Guo, Xiaoling Zheng

**Affiliations:** 1 117861 Department of Gastrointestinal Endoscopy, Fujian Provincial Hospital, Fuzhou, China; 2 117861 Fujian Medical University Provincial Clinical Medical College, Fuzhou, China; 3 117861 Department of Gastrointestinal Endoscopy, Fujian Provincial Hospital, Fuzhou, China; 4 117861 Department of Surgical Oncology, Fujian Provincial Hospital, Fuzhou, China; 5 117861 Department of Gastrointestinal Endoscopy, Fujian Provincial Hospital, Fuzhou, China; 6 117861 Department of Gastrointestinal Endoscopy, Fujian Provincial Hospital, Fuzhou, China; 7 117861 Department of Gastrointestinal Endoscopy, Fujian Provincial Hospital, Fuzhou, China

An elderly woman with persistent abdominal pain and bloating was diagnosed with obstructive sigmoid colon cancer on computed tomography (CT) ( Fig. 1 ). Because of a low oxygen saturation value caused by secondary aspiration pneumonia she was admitted to the intensive care unit; therefore transfer to a fluoroscopy-equipped operating room was inappropriate.

**Fig. 1:**
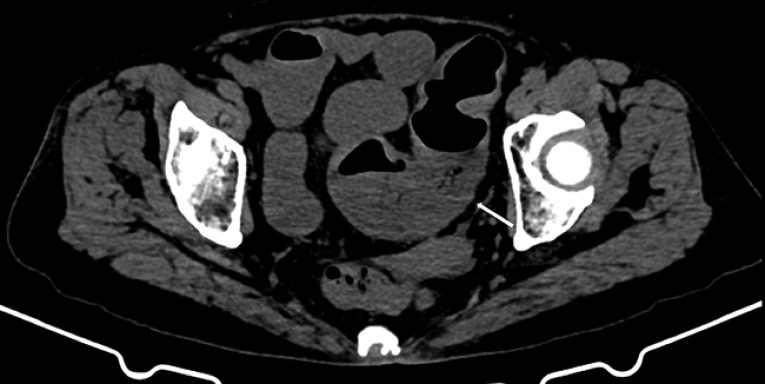
Abdominal computed tomography in an elderly woman, showing a sigmoid colon mass with distension of the distal bowel (arrow).

After multidisciplinary discussions, colonic stent placement was decided upon, using the “scope-in-scope” technique that combines digital single-operator cholangioscopy with colonoscopy. The colonoscope was used to approach the sigmoid colon, where an infiltrative mass was causing luminal narrowing ( Fig. 2 ). Then the cholangioscope (9-Fr, EyeMax; Micro-Tech, Nanjing, China) was inserted directly through the colonoscope biopsy channel.

**Fig. 2:**
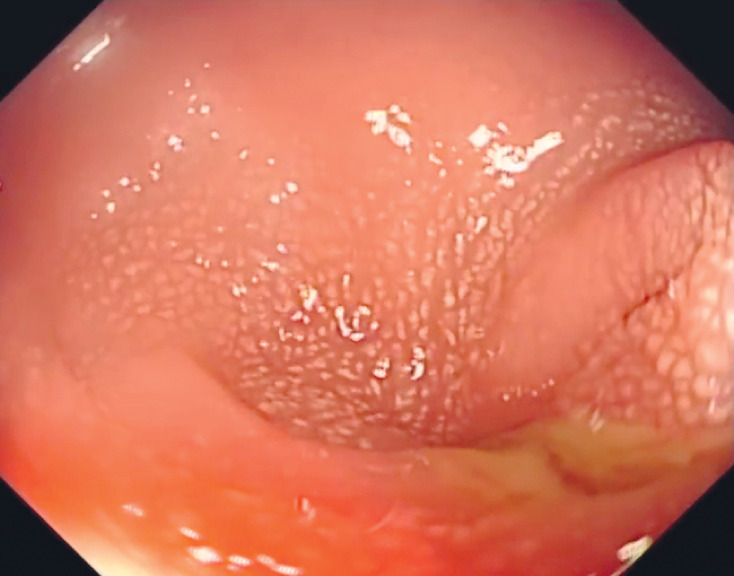
Colonoscopy showed an infiltrative mass in the sigmoid colon, accompanied by luminal narrowing.

The forward direction of the cholangioscope can be flexibly adjusted using the operating unit ( Video 1 ) and narrow lumens can be navigated under direct visualization. Thus clear observation of the internal structure of the colonic tumor was possible ( Fig. 3 ). The cholangioscope was advanced accompanied by irrigation with saline. Passage beyond the obstructed segment was confirmed when dilated intestinal lumen was seen ( Fig. 4 ).

“Scope-in-scope” technique, combining cholangioscopy and colonoscopy: swift placement of an intestinal stent without fluoroscopic guidance.Video 1

**Fig. 3:**
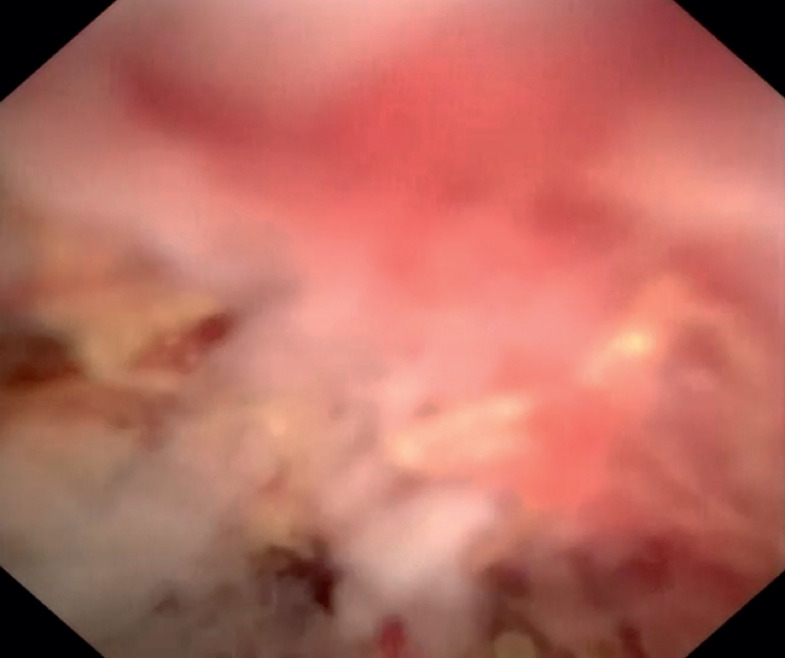
Cholangioscopy image: ulceration and necrotic areas are observed within the tumor cavity.

**Fig. 4:**
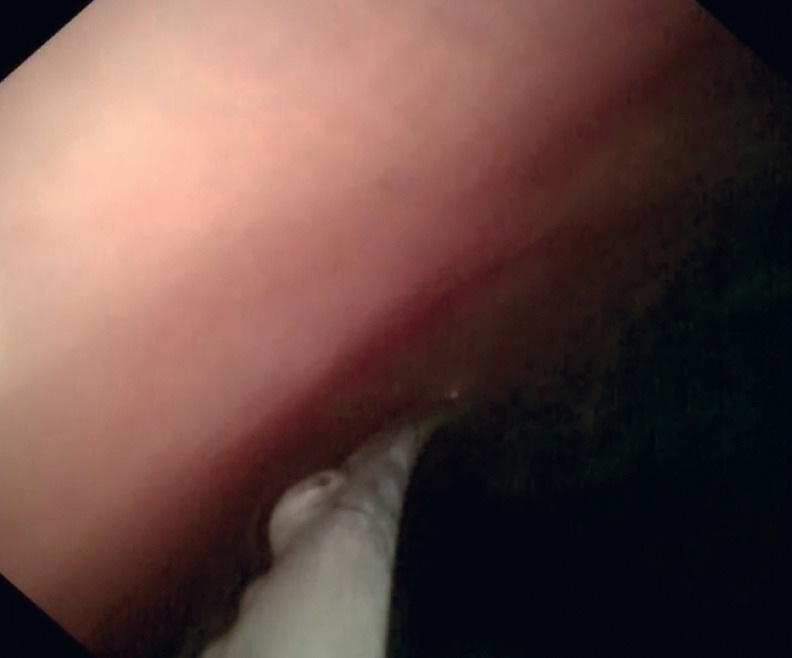
The cholangioscope showed dilated intestinal lumen, indicating passage beyond the obstructed segment.

A 0.035-inch guidewire was inserted through the forceps channel of the cholangioscope and positioned on the oral side of the tumor. The guidewire was maintained in this position as the cholangioscope was withdrawn, measuring the length of the tumor for stent selection. Guided by the wire and direct visualization, an uncoated metal intestinal stent (25 mm diameter, 9 cm length; Boston Scientific) was gradually deployed. It was possible to introduce the cholangioscope into the lumen of the incompletely expanded stent, to ensure that the stent extended beyond both ends of the narrowed segment ( Fig. 5 ).

**Fig. 5:**
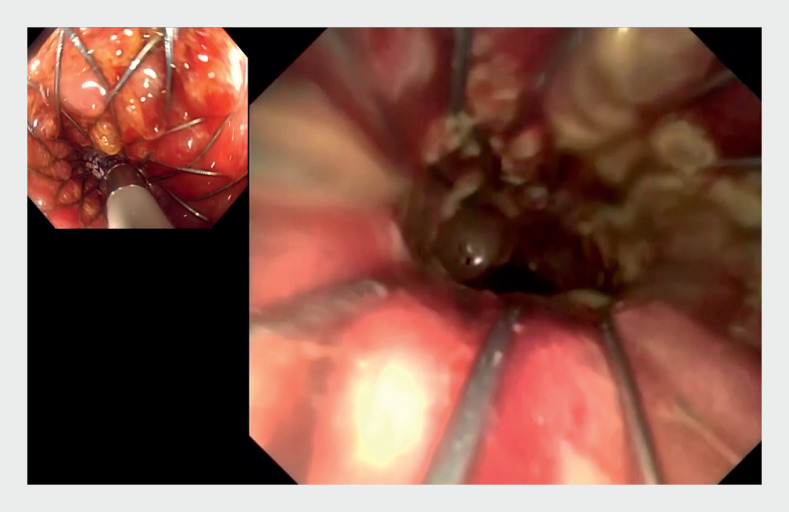
Cholangioscopy image: the cholangioscope can be introduced into the incompletely expanded stent lumen.

Postoperatively, the patient experienced significant relief from abdominal pain and bloating, bowel movements were successfully resumed, and no complications such as bleeding or perforation were encountered.

Traditional procedures for endoscopic stent placement 1 may lead to radiation exposure for both doctors and patients. This new method is particularly beneficial for certain groups, such as pregnant women, children, and patients with fragile constitutions. The “scope-in-scope” method described above, akin to its application in the appendiceal cavity 2 , uniquely allows direct observation and treatment for colonic obstructions and may reduce the risk of perforation and bleeding.

It presents a safer and more efficient alternative for stent placement in patients for whom fluoroscopy is undesirable or at institutions lacking fluoroscopic equipment.

Endoscopy_UCTN_Code_TTT_1AQ_2AF

Citation Format

Endoscopy 2024; 56: E313–E314. doi: 10.1055/a-2291-9315

## Notes

Endoscopy E-Videos https://eref.thieme.de/e-videos
*E-Videos* is an open access online section of the journal *Endoscopy* , reporting on interesting cases and new techniques in gastroenterological endoscopy. All papers include a high-quality video and are published with a Creative Commons CC-BY license. Endoscopy E-Videos qualify for HINARI discounts and waivers and eligibility is automatically checked during the submission process. We grant 100% waivers to articles whose corresponding authors are based in Group A countries and 50% waivers to those who are based in Group B countries as classified by Research4Life (see: https://www.research4life.org/access/eligibility/ ). This section has its own submission website at https://mc.manuscriptcentral.com/e-videos .
